# Responsibility without Blame for Addiction

**DOI:** 10.1007/s12152-016-9295-2

**Published:** 2017-01-07

**Authors:** Hanna Pickard

**Affiliations:** Department of Philosophy, ERI Building, University of Birmingham, Edgbaston, Birmingham, B15 2TT UK

**Keywords:** Addiction, Blame, Choice, Disease, Responsibility, Stigma

## Abstract

Drug use and drug addiction are severely stigmatised around the world. Marc Lewis does not frame his learning model of addiction as a choice model out of concern that to do so further encourages stigma and blame. Yet the evidence in support of a choice model is increasingly strong as well as consonant with core elements of his learning model. I offer a *responsibility without blame* framework that derives from reflection on forms of clinical practice that support change and recovery in patients who cause harm to themselves and others. This framework can be used to interrogate our own attitudes and responses, so that we can better see how to acknowledge the truth about choice and agency in addiction, while avoiding stigma and blame, and instead maintaining care and compassion alongside a commitment to working for social justice and good.

Drug use and drug addiction are severely stigmatised around the world.^1^ Cross-cultural studies suggest that social disapproval of addiction is greater than social disapproval of a range of highly stigmatised conditions, including leprosy, HIV positive status, homelessness, dirtiness, neglect of children, and a criminal record for burglary [1]. The 1961 UN Single Convention on Narcotic Drugs refers to drug addiction as “a serious evil for the individual” and “a social and economic danger to [hu]mankind” [2]. Our common language also expresses stigma: people who use drugs are “junkies”, mothers who use drugs are “crack moms”, and abstinence is called “getting clean” – implying, of course, that when people use drugs they are dirty. Lurid, dark images of drug use and addiction abound in the media [3].^2^ Moreover, given that possession and trafficking of many kinds of psychoactive substances are almost universally criminalised, the stigma associated with criminal offending also contributes to the stigma surrounding drug use and addiction.^3^

Stigma is a mark of social disgrace. It carries condemnation and ostracization by society and, typically, creates corresponding shame and isolation on the part of the stigmatized person.^4^ Stigma commonly impacts on the self-identity and self-esteem of drug users and addicts themselves [8] as well as presenting a psychological obstacle to seeking treatment [9]. It also has concrete practical consequences. In many parts of the world, drug addiction and drug convictions are formal barriers to healthcare, housing, benefits, employment, financial loans, and the right to vote; they may also result in long-term surveillance, forced labour, and torture and abuse during detention [10].^5^ Finally, although levels of drug use are relatively stable across different sectors of society, drug addiction and conviction rates are not equally distributed, but fall disproportionately on individuals who are otherwise vulnerable and disadvantaged, such as people who come from underprivileged socioeconomic backgrounds, have suffered from childhood abuse and adversity, struggle with mental health problems, or are members of minority ethnic groups or other groups subjected to prejudice and discrimination [10–16]. As a result, they bear a disproportionate share of the burden of the stigma and associated consequences surrounding drug use.

Why are drug users and addicts subjected to stigma and harsh treatment? No doubt a full explanation depends on a variety of complicated historical, socio-political and economic forces. But from an ideological perspective, we must also recognise how much these attitudes and policies resonate with the *moral model of addiction* which was dominant in the first half of the twentieth Century.

The moral model of addiction has two distinctive features. First, it views drug use as a choice, even for addicts. Second, it adopts a critical moral stance against this choice. Addicts are considered people of bad character with antisocial values: selfish and lazy, they supposedly value pleasure, idleness and escape above all else, and are willing to pursue these at any cost to themselves or others. In contemporary Western culture, we typically hold people responsible for actions if they have a choice and so could do otherwise, and we excuse people from responsibility if they don’t. Because the moral model of addiction sees drug use as a choice, it views addicts as responsible; because it condemns this choice, it views them as to blame – potentially deserving of the stigma and harsh treatment they in fact receive. For this reason, in so far as the moral model continues to influence – whether implicitly or explicitly – conceptions of drug use and drug addiction, the prejudice and injustices to which drug users and addicts are subjected around the world may appear justifiable.

For those who recoil from the attitudes embodied in the moral model, the *disease model of addiction* can appear by contrast to offer a desperately needed ideological corrective. Our concept of disease is not precise, and may well have different meanings and implications in different contexts of use [17]. However, with respect to models of addiction, the meaning and implication is relatively clear: “When addiction specialists say that addiction is a disease, they mean that drug use has become involuntary” [18]. According to the disease model, addiction is a chronic, relapsing neurobiological disease characterised by compulsive use despite negative consequences. Repeated drug use is supposed to change the brain so as to render the desire for drugs irresistible: the disease model maintains that addicts literally cannot help using drugs and have no choice over consumption. Compulsion can serve to explain why addicts persist in using despite the harm their use causes: if they could stop using, they would – but they can’t, so they don’t [19, 20]. But, as a result, it also removes responsibility and with it the potential for blame. The disease model of addiction can therefore serve to combat any apparent legitimacy that the stigma and harsh treatment of drug addicts might otherwise be perceived to have.^6^ With the pernicious influence of the moral model of addiction in the background, the disease model emerges as a call for compassion and a force for social justice and good.^7^

Strikingly, Marc Lewis rejects both a choice model and a disease model, offering instead what he calls a *learning model of addiction*. In his view, a choice model invites and offers to legitimate a critical moral stance: “Unfortunately, the choice model provides a convenient platform for those who consider addicts indulgent and selfish. If addiction is a choice, they reason, then addicts are deliberately inflicting harm on themselves and, more seriously, on others” [21]. But a disease model fares no better according to Lewis, for it wrongly pathologizes both the brain and the person. It wrongly pathologizes the brain because, in his view, the brain changes caused by repeated drug use are not evidence of pathology but of *neuroplasticity:* a part of the ordinary process of learning and habit formation that occurs when the brain is exposed to reward. It wrongly pathologizes the person because most addicts do not think of themselves as having a disease. Nor would it be good for them were they to do so: self-conceiving as a helpless victim of a disease and adopting the “sick role” [22] risks placing addicts in a position whereby they view themselves as dependent on medical and associated professionals for a “cure”.^8^ Yet, as Lewis emphasises, the personal experience and stories of the majority of people who have overcome their addiction to whatever degree typically involve a sense of agency and empowerment, alongside the fashioning and enacting of a life narrative that makes sense of the past while telling the story of a different future [21, 24, 25]. Crudely, addicts must come to want different things and to make different choices to overcome their addiction. Anything that helps with this task – pharmacological interventions that reduce cravings or stabilise patterns of consumption enabling gradual, monitored reduction; peer support and a sense of belonging; education and employment opportunities; the support of friends and family; books, hobbies, new pleasures; cognitive and psychological therapy; contingency management treatment; narrative self-understanding – should be used. But, to borrow a phrase from Lewis, addiction is “uncannily normal” through and through [21] – not a disease requiring specialised medical treatment, but a product of ordinary learning and development which can be overcome through further learning and development, in the form of personal growth and self-understanding.

I agree with Lewis that addiction is not a disease – at least given the typical meaning and implications of that concept. I am also sceptical that, given the state of our current understanding and evidence, we are justified in maintaining that the brain changes caused by repeated drug use are correctly classified as pathological. And I believe Lewis is correct to emphasise the central importance of a sense of agency, empowerment, and personal growth and self-understanding, in overcoming addiction. But I do not agree that we must reject a choice model of addiction.

There are two straightforward reasons why. The first is that the evidence is ever-increasing that, however hard it is for addicts to control their use, and however important it is for others to recognize and respect this struggle, addicts are not in fact compelled to use but have choice over their consumption in many circumstances. To briefly review some of this evidence: Anecdotal and first-person reports abound of addicts (including those with a DSM-based diagnosis of dependence) going “cold turkey” [13, 18]. Large-scale epidemiological studies demonstrate that the majority of addicts “mature out” without clinical intervention in their late twenties and early thirties, as the responsibilities and opportunities of adulthood, such as parenthood and employment, increase [13, 16, 26, 27]. Rates of use are cost-sensitive: indeed, some addicts choose to undergo withdrawal in order to decrease tolerance, thereby reducing the cost of future use [28]. There is increasing evidence that Contingency Management treatment improves abstinence and treatment-compliance, compared to standard forms of treatment such as counselling and cognitive-behavioural therapy, by offering a reward structure of alternative goods, such as modest monetary incentives and small prizes, on condition that addicts produce clean urine samples [29]. Experimental studies show that, when offered a choice between taking drugs or receiving money then and there in the laboratory setting, addicts will frequently choose money over drugs [30, 31]. Finally, since Bruce Alexander’s seminal experiment “Rat Park” first intimated that something similar might be true of rats [32, 33], animal research on addiction has convincingly demonstrated that, although the majority of cocaine-addicted rats will escalate self-administration, sometimes to the point of death, if no alternative goods are available, they will by contrast forego cocaine and choose alternative goods, such as saccharin or same-sex snuggling, if available [34, 35].^9^ In short, the evidence is strong that drug use in addiction is not involuntary: addicts are responsive to incentives and so have choice and a degree of control over their consumption in a great many circumstances.^10^

The second reason to maintain a choice model of addiction is that the process of overcoming addiction through a sense of agency, empowerment, and personal growth and self-understanding – a process that Lewis describes in *The Biology of Desire* [21] with great care and acuity – itself presupposes that addicts have choice and a degree of control. Agency needs to exist to be mobilized: you can only decide to quit and do what it takes to stop using and change how you live and the kind of person you are if you have some choice and control over your use and your identity. The “uncannily normal” road away from addiction is paved by ordinary moments in life where choices are made, resolve is hardened, and reflection and narrative is used to understand and buttress them. Of course, not all interventions that help addicts make changes involve choice – many things will always lie outside of our control – but choice and cognate psychological processes are nonetheless crucial elements in most if not all accounts of life changes that flow from personal growth and self-understanding, and it can be important and indeed empowering for this to be recognized and acknowledged [21, 37–39].^11^

Recall that the moral model of addiction has two features. It views drug use as a choice. And it adopts a critical moral stance against this choice. Because of the evidence that addicts respond to incentives and the role of choice and cognate psychological processes that involve agency in overcoming addiction, I believe we must accept the first feature. But that does not mean we must also accept the second. Just as addicts have choices with respect to drug use, we have choices with respect to how we respond to people who use drugs. In what follows, I offer a framework that can help us interrogate our own attitudes and responses, so that we can better see how to acknowledge the truth about choice in addiction, while maintaining care, compassion and a commitment to social justice and good. The framework derives from philosophical reflection on my personal experience working with patients whose behaviour causes them and others harm. The key is to better distinguish our concept of responsibility from our concept of blame, so that we can acknowledge agency and with it responsibility, without thereby immediately inviting let alone legitimating stigma and blame.

## Responsibility without Blame in the Clinic^12^

My first experience working in a clinical setting was in a Therapeutic Community for people with personality disorder and complex needs. Therapeutic Communities are very distinctive environments. They work by requiring genuine and sustained personal albeit professional relationships between clinicians and patients^13^ and between patients themselves. In more conventional healthcare contexts, the doctor-patient relationship is both formal and hierarchical; and there is no peer-to-peer engagement. The patient has a problem they cannot resolve on their own, and comes to the doctor for the cure. Both the doctor’s expertise, relative to the patient, and the medical nature of the clinical setting, serve to create a divide between them which can help protect doctors from personal involvement with their patients. There is both a power imbalance and a kind of emotional distance that structures the nature of the relationship and is maintained by the norms governing standard healthcare contexts. Therapeutic communities, by contrast, are informal, communal, egalitarian environments, that are committed to flattened hierarchies between clinicians and patients – all of whom are equally referred to as “community members” – where decision-making and responsibility for treatment is shared. Authenticity and emotional intimacy are central to the relationships between patients and between clinicians and patients. A great deal of time is spent together, not only in various forms of group therapy sessions, but also on everyday social activities and chores, such as cooking, eating, cleaning, gardening, or going on outings together as a community. “Community” really is the catch-word here – there is no retreat to “professional distance”.

As well as having personality disorder, many of our Community members also suffered from related conditions, such as addictions and eating disorders. Broadly speaking, these conditions are all what we might call “disorders of agency”. Core diagnostic symptoms or maintaining factors of disorders of agency are actions and omissions: patterns of behaviour central to the nature or maintenance of the condition. For instance, borderline personality disorder is diagnosed in part via deliberate self-harm and attempted suicide, reckless and impulsive behaviour, substance misuse, violence, and outbursts of anger; addiction is diagnosed via maladaptive patterns of drug consumption; eating disorders involve eating too much or too little. If a service user is to improve let alone recover from these disorders, they must change the diagnostic or maintaining pattern of behaviour [47]. For instance, service users with borderline personality disorder must stop self-harming; addicts need to quit using drugs or alcohol; anorexics must eat. There are, no doubt, equally central and important cognitive and affective components to all these disorders. Borderline personality disorder involves instability of self-image and emotional volatility; addicts may use drugs and alcohol to deal with negative emotions and psychological distress; anorexics may have over-valued ideas about low body weight and express anger and achieve a sense of control by refusing to eat. Nonetheless, actions and omissions are diagnostically central to disorders of agency: effective treatment must address these core patterns of behaviour, even if outcome is improved by an integrative approach that engages with behaviour alongside cognition and affect.

Patients with personality disorder are notoriously difficult to treat. Within psychiatry they have long been stigmatized as the patients “no one likes”. But quite generally, people who behave in ways which harm themselves or others – as addicts and those with eating disorders also do – are often very challenging for clinicians to work with effectively, as the behaviour can provoke intense emotions and reactions. Within the clinic where I worked, addictions were regularly conceptualised as forms of self-harm: unhealthy ways of coping with negative emotions and psychological distress, offering relief in the short-term, but at the cost – often itself recognised or indeed even desired by the patient – of causing long-term damage and making things worse. It is extremely difficult to see people you care for treat themselves with brutal disregard.^14^ It is also extremely difficult to see them act in ways which have a terrible impact on others, especially those who may be dependent on them and particularly vulnerable, such as their children. Clinical work requires a good therapeutic relationship. But what is the right therapeutic attitude to take when patients directly harm themselves and indirectly harm others, whether through cutting, drugs, or other means?

Within the Therapeutic Community where I worked, the clinical staff were very clear about what their attitude should be, and usually, although of course not invariably, succeeded in achieving it. Community members were responsible for their actions and omissions and accountable to the Community for them – self-harm and harm to others was not accepted – but an attitude of compassion and empathy prevailed, and they were not blamed. As a novice clinician, this stance of responsibility without blame, as I was immediately inclined to describe it, struck me forcefully. And, if I am honest, I initially had no idea how this stance was so much as conceptually possible, let alone achievable for myself within my own clinical practice. I could make sense of the idea that, despite appearances, a Community member who was, for example, misusing drugs and seriously damaging their health, their relationships, their life and the lives of those around them, might not be responsible because their addiction excused them, and hence not to be blamed. In other words, I could readily invoke a disease model of addiction, to delegitimize blame by rendering their actions involuntary and so too the possibility of attributing any responsibility to them moot. And I could make sense of the idea that, despite their addiction, they were responsible, and hence to be blamed. In other words, I could readily see how the moral model could be invoked to purportedly legitimize any blame I or others might feel. But the combination of responsibility without blame for actions that caused serious harm – to patients themselves or to others – struck me as a philosophical and clinical conundrum.

What is the source of this conundrum? Both within philosophy and within our culture at large, there is a deep-rooted tendency to link the idea of responsibility fundamentally to morality, by holding that its point or purpose is moral evaluation: the assessment of another and their behaviour as good or bad, right or wrong. In addition, such moral evaluation is sometimes understood as fundamentally affective in form [48] – embodied and expressed in our feelings towards those whose actions we morally condemn. These feelings can include anger, resentment, hate, indignation, disgust, revulsion, contempt and scorn, and are often accompanied by equally hostile thoughts and actions. At its most radical, the link between responsibility and these sorts of attitudes and expressions might be thought to be constitutive: “to regard oneself or another as responsible just is the proneness to react to them in these kinds of ways” [49]. More modestly, to hold another responsible might be understood to consist in believing that such reactions would be appropriate or fitting, even if one does not actually feel or do anything oneself [50–52]. But such nuances aside, the idea of responsibility to emerge from this picture links it fundamentally to moral evaluation via our practice of responding to others with what is in effect an affective form of blame – a set of hostile feelings typically accompanied by equally hostile thoughts and actions.^15^

Clinical practice offers a corrective to this deep-rooted tendency. Effective treatment of disorders of agency – where core symptoms or maintaining factors involve actions and omissions, including those that cause harm to the patient or to others – requires clinicians to engage with patients as responsible agents with regard to their behaviour in order to help them to change [37, 41, 46, 47]. This is because improvement or recovery from disorders of agency requires patients to break the cycle by doing things differently. As Lewis emphasises, the disease model and its corresponding “sick role” do not aid addicts in this process. After all, people will only try to change what they believe lies in their power to change [37, 53, 54]. Hence the clinical task with such patients is not to deny their agency and rescue them from blame by pathologising their behaviour, but to work with them and help them to develop their sense of agency and responsibility – to support and empower people to make different choices. In the clinic, the purpose of employing the concept of responsibility is therefore not fundamentally a form of backwards-looking moral evaluation, whereby a person is judged and potentially condemned for their past behaviour. Rather, the purpose of employing the concept of responsibility is fundamentally forwards-looking, serving to identify where there exists capacity for change thanks to the presence of choice and control, and, through clinical practices of holding responsible and to account, to motivate and encourage people to break the cycle – to develop, learn, and ultimately change what they choose to do and their sense of who they are and can be.

Of course, the exact nature of clinical practices of holding responsible and to account varies between therapeutic modalities. But within Therapeutic Communities, they typically include direct and challenging feedback, so that the negative effects of problematic behaviour on self, others and relationships is made explicit and must be faced, potentially alongside the imposition of consequences if members nonetheless continue to repeatedly engage in it (usually with advance warning and the individual’s agreement). These consequences inevitably involve a reflective component, whereby the history, circumstances, and conscious or unconscious psychological function of the problematic behaviour is explored, in order to develop a person’s own narrative self-understanding so that they can better identify what is stopping them from changing and develop a plan for how to succeed in future.^16^ But they may also involve measures that can potentially feel punitive, such as withdrawal of privileges, or time-limited suspension from the group.

It is a staple of clinical practice that, because these forms of holding responsible and to account have the potential to feel punitive, they must be effected with an attitude of concern, respect, and compassion, as opposed to being accompanied by or expressive of any of the feelings, thoughts or actions constituting an affective form of blame. For, once again, the point is not to morally evaluate and condemn, but rather to care for patients and help them improve and recover. Affective blame is understood within clinical practice to undermine the capacity of responsibility and accountability to enable change and empower, because of its propensity to make patients feel rejected, worthless, ashamed and uncared for, thereby rupturing the therapeutic relationship as well as damaging any sense of hope for the future they might otherwise have, and, correspondingly, any motivation or belief that they really can overcome their difficulties [37, 39]. The clinic thus offers a corrective to the tendency to understand our concept of responsibility as linked with affective blame, by offering a clear and established practice of attributing responsibility for problematic behaviour and holding to account without affective blame, but instead with positive regard, maintaining attitudes such as concern, respect, and compassion throughout.

Hence reflection on clinical practice brings into sharp relief a distinction between whether *the patient* has choice and a sufficient degree of control over their behaviour to be appropriately asked to take responsibility and held to account, and how *others respond* to patients who are responsible for behaviour that causes harm during the process of addressing it and holding them to account. Community members may be responsible and held to account for behaving in ways which are harmful, but without affective blame colouring the attitudes and actions of those engaging with them throughout this process.^17^

In effect, the clinical stance of responsibility without blame charts a course between the moral and disease models of addiction. On the one hand, like the moral model, it acknowledges the role of choice in addiction, thereby opening the door to the possibility of responsibility. Of course, it is important to recognise that choice and responsibility are not all or nothing, but come in degrees. People can have greater or fewer choices genuinely available to them, and more or less capacity for control. Those, like many addicts, who come from disadvantaged backgrounds typically have fewer available choices; equally, in so far as drug use is a habitual pattern of coping with negative emotions and psychological distress, the desire to use will not only be strong but equally serves an important psychological function. It is therefore extremely difficult to forego drugs unless and until the underlying feelings and difficulties are addressed, alternative healthier coping mechanisms are learned, and more options are available. For these and other reasons, agency may sometimes be diminished compared to the norm, and responsibility correspondingly reduced. But reduction is not extinction: choices may be limited and control hard to achieve, without either being nullified.^18^

On the other hand, unlike the moral model but like the disease model, the clinical stance of responsibility without blame maintains an attitude towards addicts of care. But it does not achieve this by denying addicts their agency in order to exculpate them from responsibility, thereby making any improvement or recovery dependent on a medical “cure”. Rather it aims to mobilize a sense of agency and empowerment in addicts, avoiding blame not through conceiving of them as helpless victims of disease, but through acknowledging and working with their agency without adopting moralising or stigmatising attitudes and practices. Undeniably, this runs counter to many aspects of the current cultural climate, which both appears to have a near insatiable appetite for self-righteousness and blame, and – as we saw at the opening of this article – severely stigmatises drug users and addicts. But – and this is the key point – just as those we may find ourselves unthinkingly inclined to blame and stigmatise often have a choice over their behaviour, we have a choice over how we respond. There are choices on both sides.

## In Conclusion: some First Steps towards Interrogating our Own Attitudes towards Drug Use and Addiction

Marc Lewis has diagnosed a genuine dilemma: the disease model is neither credible in the face of the evidence nor helpful in so far as it disempowers addicts; but, with the continued influence of the moral model on our thinking, a choice model invites blame and stigma by attributing agency and responsibility to addicts. In response, he has opted to distance himself from both. But that is an unstable position given the evidence that addicts respond to incentives and the importance of agency and responsibility – alongside other factors, to be sure – in overcoming addiction. We must accept a choice model of addiction – although the need to contextualise choices and understand the variety of ways control, agency, and so too responsibility, may be reduced in addiction is equally crucial [16, 19, 20, 37, 54, 56–58].^19^

However, accepting a choice model of addiction incurs a moral burden. Given that it invites blame and stigma, there is an obligation to ensure they are rejected. Choice models of addiction ought therefore to be paired with a practice of interrogating our own attitudes towards addiction alongside a commitment to working for social justice. The clinical model of responsibility without blame opens up this possibility by better distinguishing our concept of responsibility from our concept of blame, thereby helping to block any immediate tendency to slide from one to the other – in theory and in practice. But the hard task remains, of shifting attitudes and fighting for social good. I want to conclude by taking one small step towards this goal, through diagnosing how a forced choice between the moral model and the disease model functions to prevent us from reflecting on what our part is, as a society, in drug use and addiction.

Suppose we begin by asking a direct question to challenge the moral model: What precisely is supposed to be wrong with using drugs?^20^ Throughout human history, drugs have been used as means to achieve a host of valuable ends, including at minimum the following: (1) improved social interaction; (2) facilitated mating and sex; (3) heightened cognitive performance; (4) facilitated recovery and coping with stress; (5) self-medication for negative emotions, psychological distress and other mental health problems and symptoms; (6) sensory curiosity – expanded experiential horizon; and, finally, (7) euphoria and hedonia – in other words, pleasure [60]. Drugs make us feel good, provide relief from suffering, and help us do various things we want to do better. Given a commitment to basic liberal values, where individual freedom to pursue a multiplicity of goods is respected so long as harm to others does not accrue, it is difficult to see what could possibly be wrong with using drugs in and of itself [61]. In other words, as a rule of thumb, drug use becomes problematic only if it causes the negative consequences characteristic of addiction – the chronic and severe harms to self and others.^21^ For the great majority of people who use drugs, their use never escalates and gets to this point: consumption is managed so that the benefits exceed the costs, and no obvious (or unjustifiable in relation to the benefits) harms are incurred by anyone.

Suppose now we ask a further direct question: When use escalates to the point of addiction, who is to be held responsible for the ensuing negative consequences? According to the moral model, it is addicts themselves, who are not only responsible but to blame, as they are considered to be fundamentally people of bad character with antisocial values. According to the disease model, addicts are neither responsible nor to blame; their condition is the result of a disease that has taken hold, and so the negative consequences of drug use are no one’s fault – in so far as we can “blame” anything it is the disease itself.^22^ As an advocate of a choice model of addiction, I do not of course deny that some responsibility – but, crucially, responsibility as distinct from blame – lies with addicts themselves; although it is important to remember that there will sometimes be full or partial justifications or excuses, for example, those related to the need to contextualise choices and recognise how and when control may be reduced.^23^ The point I wish to emphasise however is that, in placing blame squarely on addicts or their disease respectively, both models are united in enabling us to keep the focus of our attention away from ourselves and our society, avoiding the question of whether we, as a society, also collectively bear some responsibility for drug use and addiction and their consequent harms.

Do we collectively bear such responsibility? As noted above, a disproportionate number of addicts come from underprivileged socioeconomic backgrounds, have suffered from childhood abuse and adversity, struggle with mental health problems, and are members of minority ethnic groups or other groups subjected to prejudice and discrimination. They may experience extreme psychological distress alongside a host of mental health problems apart from their addiction [12], feel a lack of psychosocial integration [10, 14, 15], and are at a socioeconomic disadvantage such that they have severely limited opportunities [10, 11, 13]. These circumstances are central to understanding addiction in many contexts [16, 19, 20, 36, 54]. Put crudely, the reason is simply that drugs offer a way of coping with stress, pain, and some of the worst of life’s miseries, when there is little possibility for genuine hope or improvement and limited alternative goods on offer. In such circumstances, whatever harms accrue from using drugs must be weighed against whatever harms accrue from not using them. For this reason, the explanation of addiction and its associated negative consequences must lie in no small part with the psycho-socio-economic circumstances that cause such suffering and limit opportunities. And the existence of these circumstances is a feature of our society for which we must all collectively take some responsibility, for we tolerate it.

Both the moral and the disease model of addiction can therefore be seen to function as a psychological defense – protecting us from focussing our attention on the existence of these circumstances and their role in explaining drug use and addiction, thereby keeping consciousness of our own collective responsibility for these facts at bay. Perhaps one reason, then, why we blame and stigmatise addicts for their choices is that it is more comfortable than facing up to aspects of our society which make drugs – whatever their costs – such a good option for many of our already vulnerable and disadvantaged members.^24^

## Footnotes

1 I include alcohol, as well as all common illicit and pharmaceutical psychoactive substances, in the reference of the term “drugs”.

2 For a project aiming to counter stereotypical media images of addicts, see Aaron Goodman’s photo documentaries of long-term heroin users: http://outcastsproject.com/ and also http://www.storyturns.org/.

3 The argument for decriminalising all psychoactive substances and regulating them instead is extremely strong [4]; see too http://www.countthecosts.org/seven-costs. Moreover, if psychoactive substances are to be criminalised, there is no valid neurochemical or public health justification for exempting alcohol, which is associated with high rates of addiction and harm [5].

4 However, it is important to note that social deviance can sometimes be a source of positive self-identity and value, especially for those belonging to a shared subculture [6, 7].

5 As I write this article in autumn 2016, there are reports that over 2500 people have been murdered in the Philippines during the past months, as part of President Rodrigo Duterte’s explicit policy to eradicate drugs by killing all those who deal or use them.

6 In *The Biology of Desire* [21] Marc Lewis suggests that this line of thought is what drives Nora Volkow, one of the powerful proponents of the disease model of addiction: “Yet her main objective [...] was to facilitate treatment for people who needed it. Calling addiction a disease not only mitigates massive volumes of stigma and guilt but also aims to provide accessible avenues for addicts to get help. This seems to be her bottom line”.

7 As Owen Flanagan puts this point: “The reclassification [of addiction as a disease and so “less voluntary, more unbidden” than a “full-on sinful life style choice”] aimed to change certain practices of blame and responsibility” [17].

8 Research suggests that this is not helpful for promoting recovery: belief in a disease model of alcoholism correlates with likelihood of relapse 6 months post-treatment [23].

9 For discussion of some of the more striking features of this research, see Pickard and Ahmed [20].

10 For further discussion, see Pickard [36], Pickard and Ahmed [20].

11 Augusten Burroughs’ take on addiction in his book *This is How: Surviving What You Think You Can’t* [40] powerfully expresses elements of both concerns, which I quote at length for a relevant first-personal perspective (but note that there will, of course, be addicts who do not share Burroughs’ perspective, as well as elements of the experience and nature of addiction that are no doubt important but omitted): “What has worked for me is to find something I wanted *more* than I wanted to drink, which was a fuck of a lot. This is less a decision than a discovery. And it’s for this reason that not everybody will get sober. My view that the way to stop drinking is to stop drinking is laughably simplistic on the surface. It’s “Just say no.” It’s also true. The way to stop drinking is to want sobriety more. And then when you feel a craving, feel the craving until it passes. But don’t act on it – any more than you wouldn’t kill somebody you feel like killing when they cut you off in traffic. Just because you want something doesn’t mean you have to have it. I know how infuriating that is to hear. Relapse is the temper tantrum that you allow yourself to have when you forbid yourself from drinking. To stop drinking, you stop drinking. You pour it out right now. Everything else – all the books, therapies, and programs – are merely hand-holding. They all strike to accomplish the same thing: to talk you into not drinking. I’m saying, if you want to stop, you will [...] To be successful at not drinking, a person needs to occupy the space in life that drinking once filled with something more rewarding than the comfort and escape of alcohol. This is the thing you have to find. You might not. Most alcoholics won’t. The truth is that people who cannot stop drinking are people who, however guilty they may feel and however dire the consequences, have become so addicted to the drug and the experience that they prefer it to the remainder of their lives. While they may truly want to be sober, they want to drink more. The thought that precedes a relapse – certainly in my case and I bet in others as well – is, “screw it”. Screw it is an idiom that means, “I no longer care”. Taking a drink is the opposite of powerlessness. It is taking firm, decisive action to terminate a state of sobriety that feels less satisfying and less convincing than drinking has felt in the past or we imagine will feel in the present. [... ]The myth that alcoholics are powerless and unable in any way to shape the outcome of their addiction is a fatal, deeply untruthful message. No alcoholic should feel powerless over alcohol. Those who die were not powerless. They either chose alcohol or they slid passively into the inevitable outcome of drinking; they made a decision by choosing to take no action. And it’s this choice that results in death [...] Ultimately, the treatment for addiction – until and if there is a successful medication – resides within the addict. You can’t spend your time waiting for rehab to “work” or for something to “fix” you. These things can – and do – inspire you or encourage you. You don’t need to take action to stop drinking. Drinking is an action: pouring the vodka into the glass, raising the glass to your lips. To stop drinking, all you have to do is sit. In 100% of the documented cases of alcoholism worldwide, the people who recovered all shared one thing in common, no matter how they did it: They didn’t to it. They just didn’t do it. You absolutely can stop drinking today, right now. The question is only, do you want to be sober more than you want to drink? Very few people can answer this question truthfully and reply, yes. I hope you’re one of them. Maybe you are. I didn’t think I was”.

12 For further discussion of this framework and related research see Pickard [37, 38, 41, 42]; Lacey and Pickard [43, 44] explore the relevance of the framework to criminal justice contexts. For some interviews and public engagement pieces, see http://www.hannapickard.com/responsibility-without-blame.html.

13 How precisely to characterise the ideal form of this relationship is a difficult question. One possibility is to draw on the distinction between care and attachment as clarified by Wonderly [45]: clinicians must care for patients without becoming attached to them; or, perhaps, by developing a form of partial or ‘quasi’ attachment only. For further discussion of the nature and efficacy of Therapeutic Communities, see Pearce and Pickard [46].

14 As family and friends who care for people with personality and related disorders of course know even better than clinicians who work with them.

15 For further discussion of affective blame see Pickard [37, 41, 42]. There is of course a great deal of variation in individual blaming style, in particular whether it is ‘hot’ and direct in its aggressiveness or ‘cold’ and passive-aggressive.

16 For this reason, although the fundamental purpose of employing the concept of responsibility in the clinic is forwards-looking, the practice of doing so yet contains some backwards-looking elements, in so far as reflective exploration of the past can be used to facilitate change in the future. For discussion see Pickard [24].

17 I am of course not claiming that clinicians and community members always manage to achieve this stance of responsibility without blame in practice. Rather, it is a guiding norm or implicit principle of clinical engagement in certain therapeutic contexts, which is often if not inevitably achieved. For discussion of some of the factors and techniques facilitating the clinical skill of holding people responsible without blame, see Pickard [37], Pearce and Pickard [46, 54], Lacey and Pickard [43, 44].

18 The concept of responsibility employed in the clinic is linked to choice and control – perhaps because of the power of these ideas to motivate and enable a sense of agency for change [38, 39]. But for those who prefer a reasons-responsive account of responsibility [52], it can be substituted in theory and the basic distinction between responsibility and blame preserved: the key is simply not to link an account of responsibility to the emotions and attitudes constitutive of affective blame. For discussion of the idea of degrees of responsibility in relation to a reasons-responsive account, see Coates and Swenson [55]. For discussion of how potentially to understand the ways in which choice and control may be present yet reduced in addiction, see Holton and Berridge [56] and Henden [57].

19 Note that Lewis himself emphasises the importance of these considerations in relation to choice throughout *The Biology of Desire* [21], both tacitly in his recounting of the personal stories of addicts, and explicitly in his introductory and concluding discussions (see especially chapters one, eight and nine). For this reason, although he purports to be hostile to traditional choice models, it is possible he would not be hostile to a more nuanced choice model thus reconfigured.

20 Recall that I count alcohol, as well as all common illicit and pharmaceutical psychoactive substances, as drugs (see footnote 1); and that there is no valid neurochemical or public health justification for distinguishing alcohol and many such pharmaceuticals from illicit drugs (see footnote 3) – indeed, in some cases (e.g. methamphetamine and d-amphetamine [59]) there is little neurochemical or experienced difference between common pharmaceuticals and illicit drugs.

21 “As a rule of thumb” because there are of course exceptions, most poignantly, the tragic cases where typically young and inexperienced recreational users die due to using drugs cut with toxins, mixing multiples substances, or taking too high a dose. However, this risk could be significantly reduced, through decriminalization and regulation ensuring that reliable sources of drugs were available, alongside adequate public education initiatives providing accurate information on how to use drugs safely. Carl Hart has argued forcefully for both points and offers drug safety education on his website; see http://drcarlhart.com/.

22 Although note for clarity that it is of course consistent with a disease model to hold that despite not being responsible for current drug use and its consequences, addicts nonetheless may bear some responsibility for becoming addicted in the first place.

23 For further discussion of these and related points in relation to criminal responsibility, see Morse [62].

24 Thanks to Steve Matthews and Anke Snoek for helpful comments on a previous version of this article, and to Stephen Morse for introducing me to Augusten Burroughs’ work, quoted in footnote 11.
